# Macrophages and immunometabolism in diminished ovarian reserve: pathological features and therapeutic implications

**DOI:** 10.3389/fendo.2026.1883984

**Published:** 2026-10-05

**Authors:** Bingxia Li, Jia Zhang, Quansheng Wu, Xiaohua Zhang

**Affiliations:** Gansu University of Chinese Medicine, Clinical School of Traditional Chinese Medicine, Lanzhou, China

**Keywords:** diminished ovarian reserve, fibrosis, inflammaging, macrophages, metabolic reprogramming

## Abstract

Diminished ovarian reserve (DOR) is a prevalent reproductive endocrine disorder that significantly impairs female fertility. While traditional research has emphasized intrinsic oocyte defects, growing evidence highlights the ovarian immune microenvironment—specifically macrophages—as a critical regulator of follicular fate and reserve maintenance. Nevertheless, the mechanisms by which macrophage dysfunction drives DOR remain incompletely understood, and the interplay between chronic inflammation and macrophage metabolic reprogramming has yet to be systematically elucidated. This narrative review synthesizes literature from immunology, metabolism, and reproductive medicine, framing current evidence as established findings, putative mechanisms, and emerging hypotheses. The key findings are threefold: 1) macrophage polarization imbalance, which is marked by sustained M1 activation and impaired M2 function, constitutes a core pathological feature of DOR; 2) this imbalance is driven by metabolic reprogramming—specifically, metabolic inflexibility in M1 (persistent glycolytic commitment) and suppressed oxidative phosphorylation in M2—which links inflammaging to ovarian fibrosis and follicular depletion; and 3) pharmacological modulation of macrophage immunometabolism (e.g., AMPK/PPARγ activators, LXR agonists, and itaconate) and engineered delivery platforms (e.g., exosomes and targeted nanocarriers) hold preclinical promise. This review establishes an “immunometabolic axis” framework that integrates macrophage polarization, metabolic dysregulation, and ovarian reserve loss, advancing the understanding of DOR pathogenesis and informing precision interventions.

## 1 Introduction

Diminished ovarian reserve (DOR) is a reproductive endocrine disorder characterized by reduced oocyte quantity and quality, manifesting as menstrual irregularities, reduced fertility, and perimenopausal symptoms (1). Early studies have focused primarily on intrinsic oocyte defects, including genetic mutations, mitochondrial dysfunction, and telomere attrition. However, accumulating evidence highlights the ovarian microenvironment as a critical regulator of follicular fate (2). This microenvironment comprises diverse cellular and structural components—including granulosa cells, theca cells, vascular endothelial cells, fibroblasts, and immune cells—embedded within a dynamic extracellular matrix (ECM) (3). This milieu provides physical support and nourishment for oocytes (4) and serves as a critical signaling hub (5). It integrates endocrine, paracrine, and autocrine signals to coordinate follicular recruitment, dominance selection, oocyte maturation, and ovulation (6). Endocrine inputs include pituitary-derived follicle-stimulating hormone (FSH) and luteinizing hormone (LH) (7) and gonadal steroid feedback. Paracrine signals comprise oocyte-secreted BMP-15 and GDF-9 (8), granulosa-derived activin (9) and IGF-I, and theca-derived bone morphogenetic proteins (BMPs) and androgens. Autocrine regulation involves intra-granulosa insulin-like growth factor (IGF)/IGF-binding protein (IGFBP) signaling and brain-derived neurotrophic factor (BDNF)–tropomyosin receptor kinase B (TrkB) autoregulation (10).

Among the immune constituents of this microenvironment, macrophages are the most abundant innate immune cells (11) and exhibit marked plasticity, dynamically transitioning between the pro-inflammatory (M1) and anti-inflammatory/repair (M2) phenotypes in response to local stimuli (12) such as inflammatory cytokines, metabolic stressors (e.g., hypoxia or oxidative stress), and clearance signals from apoptotic cells. However, under pathological conditions or during ovarian aging, the microenvironment undergoes remodeling characterized by persistent low-grade inflammation and progressive fibrosis (13). Evidence from *in vitro* models of ovarian cell senescence and murine aging studies suggests that mitochondrial damage-associated molecular patterns (mtDAMPs) (14), pro-inflammatory cytokines, and senescence-associated secretory phenotype (SASP) factors serve as critical initiators of this inflammatory cascade (15), promoting M1 macrophage polarization and disrupting M1/M2 homeostasis (16, 17). This imbalance may accelerate follicular reserve depletion through multiple mechanisms, including induction of oxidative stress, fibrosis and vascular abnormalities, and disruption of primordial follicle pool homeostasis (18).

Macrophage metabolic reprogramming couples microenvironmental perturbations to cellular dysfunction. This process is characterized by glycolytic inflexibility and lipid metabolic dysregulation, encompassing impaired mitochondrial fatty acid oxidation (FAO), aberrant lipid uptake and accumulation, and cholesterol efflux dysfunction that promotes foam cell formation (19, 20). Consequently, metabolic and polarization disturbances establish a self-reinforcing feedback loop: shifts in polarization drive metabolic reprogramming, while metabolic disturbances reciprocally perpetuate polarization imbalance. The metabolic-to-polarization axis of this loop is mediated by specific intermediates acting as direct signaling molecules. Succinate stabilizes hypoxia-inducible factor 1-alpha (HIF-1α) to sustain glycolysis and interleukin-1 beta (IL-1β) expression (21), whereas citrate-derived acetyl-CoA promotes histone acetylation to license inflammatory gene transcription. Collectively, these mechanisms transcriptionally reinforce M1 identity. Conversely, an enhanced oxidative metabolism repolarizes the M1 macrophages toward M2 (22), and glycolytic inhibition attenuates inflammation (23). These findings support the notion that the metabolic state is an active determinant, not merely a passive reflection, of polarization fate (24). Within this remodeled microenvironment, persistent inflammatory and fibrotic cues impose metabolic constraints on resident macrophages. We therefore posit that macrophage metabolic reprogramming represents the pivotal mechanism underlying polarization imbalance, thereby driving the pathogenesis of DOR (25).

To support this hypothesis, we summarize the homeostatic roles of macrophages in normal ovarian physiology and their alterations in DOR, examine the interplay between polarization imbalance and metabolic reprogramming, and discuss mechanisms driving follicular exhaustion. We then review the therapeutic strategies targeting macrophage immunometabolism and outline future directions. Integrating advances in immunology, metabolomics, and reproductive medicine, this review presents an immunometabolic perspective on the pathogenesis of DOR to guide precision interventions within the ovarian microenvironment.

### 1.1 Scope and methodology

This is a narrative review. Literature was identified through targeted searches of PubMed, Web of Science, and Embase using combinations of the following keywords: “diminished ovarian reserve”, “premature ovarian insufficiency”, “ovarian aging”, “macrophage polarization”, “immunometabolism”, “inflammaging”, “metabolic reprogramming”, and “ovarian fibrosis”. Additional studies were retrieved through manual review of the reference lists and forward citation tracking of key publications. Studies were selected based on relevance to the immunometabolic mechanisms of macrophage dysfunction in ovarian reserve decline, with emphasis on mechanistic studies, preclinical models, and recent clinical evidence. No formal risk-of-bias assessment or meta-analysis was performed. As a narrative review, this work was not registered with PROSPERO and does not adhere to the PRISMA guidelines.

## 2 Dual roles of macrophages in ovarian homeostasis

### 2.1 Macrophage origin, heterogeneity, and plasticity

Macrophages are essential components of the ovarian microenvironment. They originate from two distinct sources: embryonic yolk sac progenitors that establish self-renewing tissue-resident populations and bone marrow-derived monocytes that are recruited in response to injury or inflammation (26, 27). These cells are distributed throughout the ovary—including the follicular theca, corpus luteum, stroma, and atretic follicles—and constitute one of the most abundant leukocyte populations in the organ (28).

Macrophages exhibit remarkable functional plasticity, enabling them to adopt distinct or even opposing phenotypes in response to local signals (29). This polarization exists along a continuum rather than as a strict binary classification. Classically activated M1 macrophages are induced by interferon gamma (IFN-γ) and lipopolysaccharides (LPS) and promote a pro-inflammatory state characterized by tumor necrosis factor alpha (TNF-α), IL-1β, and reactive oxygen species (ROS) production, which are essential for host defense (30). In contrast, M2 macrophages are driven by anti-inflammatory cytokines such as IL-4, IL-13, and IL-10 and mediate tissue repair through the secretion of IL-10, expression of arginase-1, and the promotion of angiogenesis and matrix remodeling (31, 32). This M2 phenotype encompasses several subsets: M2a cells (IL-4 and IL-13) (33), M2b cells [immune complexes and Toll-like receptor (TLR) ligands] (34), M2c cells (glucocorticoids and IL-10), Mox macrophages (oxidized phospholipids via Nrf2) (35), and tumor-associated M2d macrophages (adenosine receptor ligands). This phenotypic spectrum is dynamic and responsive to microenvironmental cues.

### 2.2 Role of macrophages in the ovarian cycle

#### 2.2.1 Follicular development and atresia

During early folliculogenesis, macrophages promote follicular development through paracrine factors including hepatocyte growth factor, epidermal growth factor, and basic fibroblast growth factor (36). During follicular atresia, endothelium-derived IL-33 recruits macrophages to clear apoptotic debris and preserve tissue integrity. IL-33 deficiency impairs this clearance, accelerating ovarian aging and shortening the reproductive life span (37, 38).

#### 2.2.2 Ovulation

Ovulation is an acute inflammatory event triggered by a surge in LH, which recruits macrophages and neutrophils to the preovulatory follicle (39). The pro-inflammatory cytokines from these cells, including TNF-α and IL-1β, promote prostaglandin synthesis and matrix metalloproteinase activation, thereby facilitating follicular rupture (40).

#### 2.2.3 Luteal formation and regression

Following ovulation, macrophages infiltrate the developing corpus luteum, promoting angiogenesis and progesterone production (41). During luteal regression, they clear apoptotic luteal cells via efferocytosis, which is dependent on phosphatidylserine recognition and integrin signaling (42). Beyond their cyclic functions, macrophages preserve ovarian reserve through two distinct mechanisms. First, efficient efferocytosis prevents the accumulation of pro-inflammatory debris (43). Second, M2-polarized macrophages suppress the PI3K/AKT/mTOR pathway to maintain primordial follicle dormancy (44). Thus, macrophage dynamics across the ovarian cycle are fundamental to both reproductive cyclicity and long-term reserve maintenance.

## 3 Inflammaging drives macrophage polarization imbalance

### 3.1 DOR immunometabolism: inflammatory aging and fibrosis

Under ovarian aging or pathological conditions, the local microenvironment shifts from homeostasis to a pro-fibrotic, pro-inflammatory state, characterized by chronic low-grade inflammation, excessive collagen deposition and cross-linking (45), elastin fragmentation and reduced fibronectin and hyaluronan levels (46), and progressive tissue stiffening (47). Accumulating evidence indicates that this microenvironmental reprogramming drives macrophage polarization toward a pro-inflammatory phenotype. Mechanistically, sustained exposure to pro-inflammatory cytokines, hypoxia-inducible factors (48), and ECM-derived DAMPs (49) promotes metabolic reprogramming, shifting bioenergetics from oxidative phosphorylation (OXPHOS) toward glycolysis and driving mitochondrial dysfunction and ROS overproduction (23). This metabolic shift represents a pivotal mechanism linking microenvironmental cues to polarization imbalance (50, 51), thereby driving DOR pathogenesis (52, 53).

The core pathological backdrop for these processes is “inflammaging”—defined as an age-related, chronic, low-grade systemic inflammation that manifests locally in the ovarian microenvironment (54). This state is marked by persistently elevated pro-inflammatory cytokines (e.g., IL-6, TNF-α, and IL-1β) and increased oxidative stress (55). Ovarian inflammaging is sustained by chronic local inflammation arising from multiple factors, including senescent cell accumulation (with SASP), mitochondrial dysfunction, endoplasmic reticulum stress, persistent antigenic stimulation, and dysbiosis-related systemic inflammation (56). This inflammatory milieu drives both macrophage metabolic reprogramming and progressive tissue fibrosis, the latter mediated largely by transforming growth factor beta (TGF-β) signaling (57). Indeed, sustained TGF-β signaling stimulates ovarian cortical and medullary fibroblasts to differentiate into highly secretory myofibroblasts, resulting in excessive deposition of ECM components such as type I and type III collagen (58). This fibrotic process fundamentally alters the biophysical and biochemical properties of ovarian tissue: tissue stiffness increases and compliance decreases, physically constraining the space available for primordial and antral follicles, while angiogenesis is severely impaired, diminishing the delivery of nutrients, gonadotropins, and oxygen (59, 60).

The resulting ischemic, hypoxic, and signaling-deficient microenvironment impairs granulosa cell function and disrupts oocyte energy metabolism and meiosis, thereby reducing follicular developmental potential and accelerating atresia (61). Moreover, inflammaging and fibrosis are not independent: they form a self-reinforcing feedback loop in which inflammatory mediators perpetuate fibrotic progression, while the stiff fibrotic matrix amplifies inflammatory signaling through mechanical cues (62). This pathological network underlies the dysfunctional microenvironment characteristic of ovarian reserve decline, highlighting critical targets for therapeutic intervention (63).

### 3.2 Macrophage polarization imbalance in DOR

Chronic exposure to this pathological microenvironment drives a marked shift in ovarian macrophage polarization toward a pro-inflammatory, dysfunctional phenotype.

#### 3.2.1 Excessive M1 macrophage activation

In aging mouse ovaries and chemotherapy-induced injury models, classical M1 markers [e.g., inducible nitric oxide synthase (iNOS), TNF-α, and IL-6] are significantly elevated, providing direct evidence of M1 hyperactivation (64). Mechanistically, cathepsin B expression is markedly upregulated in ovarian macrophages during aging. This protease activates the nuclear factor kappa B (NF-κB) pathway, driving the secretion of pro-inflammatory cytokines, including TNF-α (65, 66). We posit that this cathepsin B–NF-κB axis bridges inflammatory aging and M1 polarization, consistent with histopathological evidence linking chronic inflammation to age-related ovarian dysfunction. However, direct immunoprofiling of human DOR ovarian tissue remains limited, with the majority of evidence derived from peripheral blood or animal models that may not reflect the local microenvironment (67, 68). This discrepancy highlights an urgent need for direct immunophenotypic characterization of macrophage polarization within the ovarian cortex of patients with DOR.

#### 3.2.2 Impaired and dysregulated M2 macrophages

Firstly, M2 macrophage phagocytic clearance is markedly impaired. Macrophages from DOR follicular fluid or aging animal ovaries exhibit reduced phagocytosis of apoptotic cells, which is linked to downregulated clearance receptors such as Mer tyrosine kinase (MerTK) and impaired degradation of apoptotic debris (67, 69). This defect leads to apoptotic remnant accumulation, perpetuating chronic inflammation in the ovarian microenvironment (70). Secondly, the M2 subset becomes functionally imbalanced: pro-fibrotic M2a macrophages expand, while immunomodulatory M2c cells are suppressed (17, 71). Although the overall M2 marker expression may remain unchanged, the pool shifts from a reparative to a pro-fibrotic phenotype, as evidenced by M2-mediated collagen deposition into the ECM and the consequent ovarian fibrosis (72).

#### 3.2.3 Pathological macrophage morphologies

Beyond classical M1/M2 polarization, the aging ovary harbors distinct pathological macrophage morphologies, including multinucleated giant cells (73) and lipid-laden foam cells (74). These cells reflect profound metabolic dysregulation and are closely associated with local lipid metabolic disturbances and chronic inflammatory signaling in ovarian tissue (75) (**Figure 1**).

**Figure 1:**
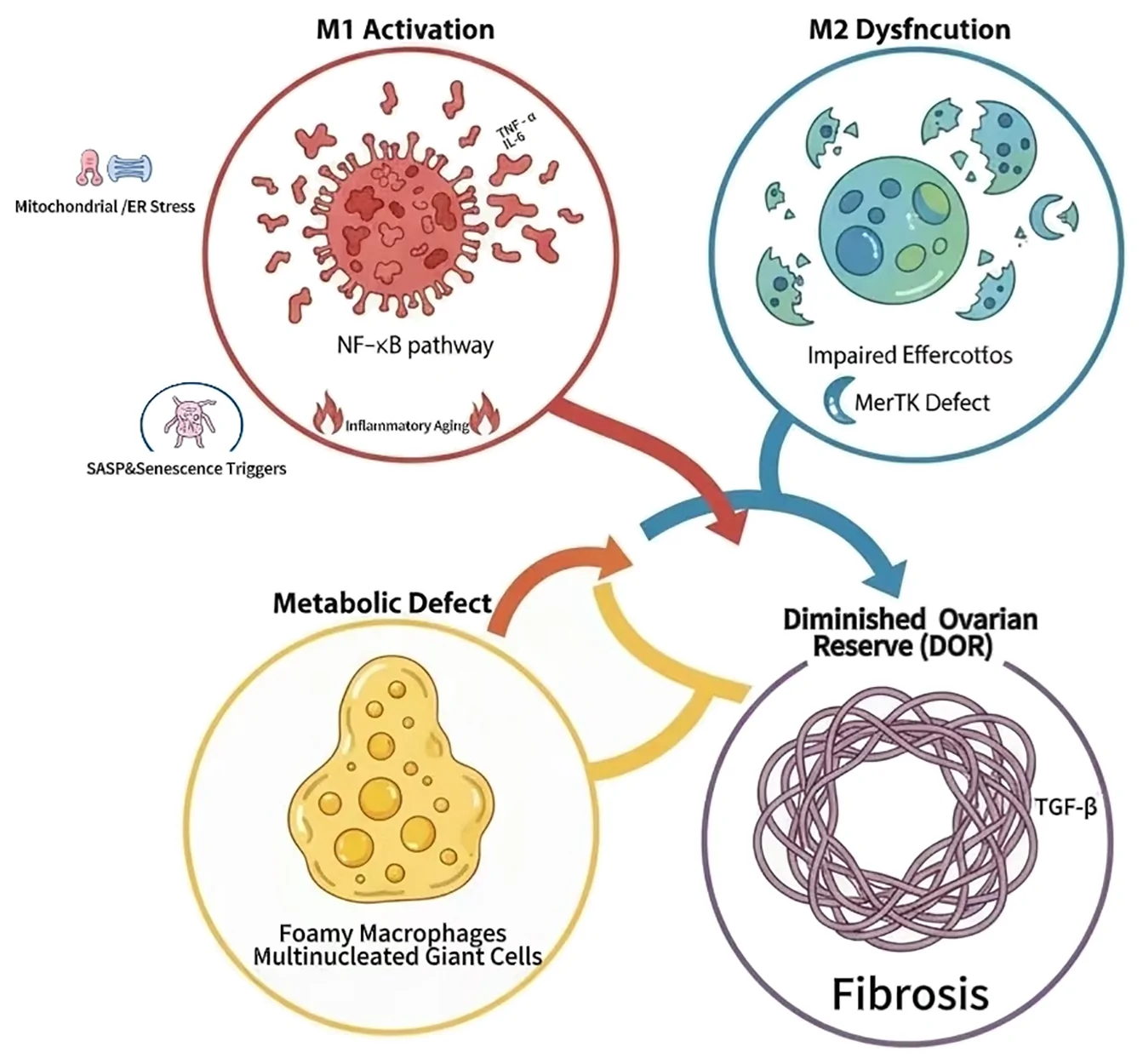
Schematic illustration of macrophage-mediated inflammatory stress and fibrosis in diminished ovarian reserve (DOR). In the aged or pathological ovarian microenvironment, chronic low-grade inflammation (inflammaging) drives M1 macrophage polarization via nuclear factor kappa B (NF-κB) activation, accompanied by mitochondrial and endoplasmic reticulum stress and the senescence-associated secretory phenotype (SASP). Concurrently, M2 macrophages exhibit functional impairment, characterized by defective phagocytosis and loss of Mer tyrosine kinase (MerTK) signaling, which compromises apoptotic cell clearance. These processes collectively promote the formation of foamy macrophages and multinucleated giant cells, amplify transforming growth factor beta (TGF-β) signaling, and ultimately drive ovarian fibrosis and follicular reserve depletion.

## 4 Core driver: metabolic reprogramming of macrophages

### 4.1 Mitochondria: the central hub of macrophage metabolic reprogramming

The mitochondria in macrophages serve dual roles as energy-producing organelles and critical nodes coupling metabolic reprogramming to functional polarization (76). The metabolic state of the mitochondria, which is reflected by OXPHOS activity (77), ROS generation (78), and mitochondrial dynamics, critically determines macrophage polarization. Within the DOR framework, macrophage metabolic reprogramming intrinsically drives polarization imbalance (79), functional decline (16), and, ultimately, ovarian microenvironmental deterioration (64).

#### 4.1.1 M1 macrophages: glycolysis-driven inflammation

Activated M1 macrophages maintain high glycolytic rates even in the presence of oxygen, generating lactate rather than engaging mitochondrial OXPHOS (80, 81). This metabolic profile damages the mitochondria, increasing mitochondrial ROS (mtROS), altering membrane potential, and impairing electron transport chain (ETC) activity. Consequently, the dysfunctional mitochondria generate mtROS and release mitochondrial DNA (mtDNA), which activate the NLRP3 inflammasome and cGAS-STING pathways (82).

M1 polarization disrupts the key enzymatic steps in the tricarboxylic acid (TCA) cycle, particularly isocitrate dehydrogenase and succinate dehydrogenase (83), leading to the accumulation of metabolic intermediates. Citrate is transported to the cytosol, where it serves as a precursor for nitric oxide and pro-inflammatory lipid mediators such as prostaglandin E_2_. On the other hand, succinate accumulation stabilizes HIF-1α and enhances IL-1β transcription (84). This succinate–HIF-1α–IL-1β axis represents a critical signaling pathway that links metabolic reprogramming to inflammation (50). Concomitantly, OXPHOS and FAO are suppressed in M1 macrophages (85). Collectively, these metabolic changes render M1 macrophages metabolically active yet tissue-destructive inflammatory effectors.

The glycolytic shift facilitates rapid ATP generation and provides biosynthetic precursors through the pentose phosphate pathway and glycolytic intermediates, thereby supporting the nucleotide, amino acid, and fatty acid synthesis required for the production of pro-inflammatory cytokines (e.g., TNF-α, IL-6, and IL-1β) and ROS (86).

#### 4.1.2 M2 macrophages: oxidative phosphorylation in tissue repair

In contrast to the pro-inflammatory M1 macrophages, M2 cells—particularly the M2a and M2c subsets associated with tissue repair and immunomodulation—exhibit a metabolic profile dominated by OXPHOS and FAO (87). These cells maintain intact, functional mitochondrial networks that efficiently oxidize fatty acids (via β-oxidation) and glutamine to fuel the TCA cycle and ETC, generating ample ATP (88). Mitochondrial integrity is essential for tissue repair: an impaired dynamics, whether from dysregulated fusion/fission or defective mitophagy, attenuates M2 polarization and promotes a pro-inflammatory shift (89). The OXPHOS-dominant program fuels energy-demanding processes including phagocytosis, chemotaxis, and synthesis of anti-inflammatory cytokines (e.g., IL-10 and TGF-β) and reparative ECM proteins. FAO is indispensable for IL-4/IL-13-driven M2 polarization, and blockade of this pathway impairs alternative activation and tissue repair (90).

### 4.2 Metabolic dysregulation in macrophages in DOR

#### 4.2.1 Metabolic rigidity and functional impairment

The ability of macrophages to dynamically switch between pro-inflammatory glycolysis and reparative OXPHOS in response to microenvironmental cues is essential for maintaining immune homeostasis (91). In the persistent inflammatory milieu of DOR, chronic stimulation by TNF-α, IL-1β, and DAMPs (67) induces epigenetic reprogramming, including histone acetylation and the methylation changes that lock macrophages in a glycolysis-dominant state (92). This metabolic inflexibility impairs their response to reparative cytokines such as IL-4/IL-13, preventing the transition to OXPHOS and compromising functional plasticity. Macrophages derived from the follicular fluid of patients with DOR exhibit a markedly reduced phagocytic capacity (93). Moreover, the reduced mitochondrial membrane potential in these cells indicates a broader bioenergetic deficit. These deficiencies in metabolic adaptability impair the macrophage-mediated phagocytic clearance and tissue repair, perpetuating a cycle of apoptotic debris accumulation, chronic inflammation, and tissue damage within the ovary.

#### 4.2.2 Macrophage lipid dysregulation and foamy transformation

Lipid metabolic disruption and foamy transformation in DOR-associated macrophages reflect an immunometabolic imbalance in the ovarian microenvironment (94). Metabolomic profiling of follicular fluid from patients with DOR revealed marked alterations in glycerophospholipid and cholesterol/sterol metabolism (95). Altered levels of specific metabolites, including phosphatidylcholine, drive macrophage polarization toward a pro-inflammatory M1 phenotype, thereby promoting lipid dysregulation (96).

Single-cell sequencing has identified a distinct macrophage subpopulation in aging ovarian tissue characterized by a C–X3–C motif chemokine receptor 1-low, CD81-high (Cx3cr1^low^Cd81^hi^) signature with an elevated peroxisome proliferator-activated receptor gamma (PPARγ) expression (97). The upregulation of PPARγ likely represents an early compensatory response to microenvironmental lipid overload, aimed at enhancing the lipid-handling capacity. However, this compensatory mechanism ultimately fails under persistent inflammatory and oxidative stress associated with DOR. Accelerated follicular atresia releases lipid-rich substrates, including apoptotic debris and oxidized low-density lipoproteins, while inflammatory signaling suppresses key transcriptional regulators such as liver X receptors (LXRs) (98). Consequently, expression of the cholesterol efflux transporters ATP-binding cassette transporters A1 and G1 (ABCA1/ABCG1) declines (99). This imbalance between lipid uptake and efflux drives the intracellular accumulation of cholesterol esters and triglycerides, culminating in the formation of lipid-laden foamy macrophages (100).

These foamy macrophages display a terminally dysregulated phenotype characterized by an increased IL-1β secretion, a decreased IL-10 production, an impaired chemotaxis and phagocytosis, and the promotion of fibrosis (101). A 2025 study demonstrated their pathogenic involvement: CD68^+^BODIPY^+^ foamy macrophage clusters in ovarian stroma correlated positively with fibrotic burden and atretic follicle number. Furthermore, the conditioned medium from these macrophages directly induced the secretion of collagen and the proliferation of ovarian stromal fibroblasts *in vitro* (102).

Mitochondrial dysfunction initiates macrophage metabolic reprogramming in DOR, driving M1 polarization and ovarian reserve depletion (16). The transition from PPARγ-mediated compensation to lipid collapse, accompanied by foamy macrophage formation, traces the pathogenic trajectory of the ovarian immunometabolic microenvironment (103, 104). Within this framework, mitochondrial damage serves as the trigger, metabolic rigidity as the driver (105), and foam cells as the effector (106). These interrelated components constitute a self-reinforcing cycle that progressively depletes the ovarian reserve. Therefore, restoring mitochondrial homeostasis and metabolic flexibility in macrophages represents a critical therapeutic strategy to disrupt this cascade and preserve ovarian function (107, 108).

## 5 Mechanisms of ovarian reserve depletion

Macrophages with polarization imbalance compromise the ovarian reserve through intertwined pathways.

### 5.1 Inflammatory and oxidative damage to follicles

Activated M1 macrophages are the primary effector cells driving local ovarian inflammation and oxidative injury. During ovarian aging or persistent oxidative stress (86), damaged and apoptotic cells release DAMPs, including mtDNA and HMGB1. These DAMPs chronically activate TLR9 and the NLRP3 inflammasome in macrophages (109), thereby promoting classical M1 polarization. This polarization state is characterized by persistent NF-κB and STAT1 signaling, which sustains local production of TNF-α and IL-1β and expression of iNOS.

Mechanistic studies across diverse inflammatory models indicate that these mediators establish a self-sustaining autocrine/paracrine feedback loop: TNF-α and IL-1β further activate NF-κB signaling in neighboring macrophages (110). In addition, M1 macrophages generate ROS via NOX2 and reactive nitrogen species (RNS) via iNOS (111), producing peroxynitrite (ONOO^−^) that inflicts metabolic and genotoxic damage on follicular cells. Metabolically, ROS/RNS impair mitochondrial respiratory complexes I and IV, suppress OXPHOS, and reduce ATP synthesis. These events trigger additional mitochondrial ROS production, establishing a self-sustaining ROS–RNS cycle that disrupts the energy homeostasis in oocytes and granulosa cells (112). Genotoxically, RNS such as ONOO^−^ induce DNA strand breaks and base modifications (e.g., 8-nitroguanine), impair base excision repair enzymes (e.g., APE1), and drive granulosa cell apoptosis through p53-dependent pathways (113).

The inflammatory–oxidative/nitrosative stress cycle promotes follicular atresia and ovarian reserve decline (114). Within growing follicles, M1 macrophage-derived IL-6 activates the JAK/STAT3 pathway in granulosa cells, amplifying local inflammatory responses and sensitizing these cells to apoptosis (115). Concurrently, IL-1β downregulates FSH receptor expression and inhibits CYP19A1 (aromatase) activity. These effects diminish the follicular responsiveness to gonadotropins and impair estrogen synthesis, ultimately compromising follicle maturation and selection (32).

### 5.2 Fibrosis and microenvironmental dysregulation

In the inflammatory microenvironment associated with ovarian aging or injury, macrophage polarization is skewed toward M1 activation and the pro-fibrotic M2a subtype. This imbalance is a major driver of fibrosis. Studies in aging ovarian models consistently demonstrate that these macrophages are significant sources of pro-fibrotic mediators, including TGF-β and platelet-derived growth factor-BB (PDGF-BB) (116). TGF-β is the principal regulator of fibrosis: it drives the differentiation of ovarian stromal fibroblasts into myofibroblasts via the Smad2/3 pathway and enhances the production of the ECM (117). As a potent mitogen, PDGF-BB further promotes fibroblast proliferation, migration, and collagen synthesis (61, 118).

These biomechanical changes are active rather than passive. Mechanobiology research demonstrates that dense collagen networks transmit abnormal mechanical signals to follicular cells, particularly granulosa cells, via integrin–FAK signaling (119). In response to ECM stiffening (120), these cues activate downstream pathways that reorganize the cytoskeleton and alter the gene expression and cell fate. Such perturbations disrupt the intercellular communication between oocytes and granulosa cells, generating a microenvironment that compromises follicle survival and development (121).

Fibrosis and vascular dysfunction are interconnected processes that compromise follicular nourishment (25). An M1 macrophage-dominated inflammatory environment suppresses pro-angiogenic factors, including vascular endothelial growth factor A (VEGF-A), while upregulating endogenous anti-angiogenic mediators (122, 123). TNF-α and other inflammatory mediators further damage endothelial cells, disrupt tight junctions, and impair neovascularization. Recent animal studies indicate that dysfunctional macrophages destabilize the existing vasculature, resulting in reduced perivascular cell coverage, endothelial cell loss, hemorrhage, and follicular degeneration (124). Destruction of the vascular network subsequently initiates a cycle of ischemia, hypoxia, and oxidative stress in the ovary (16). Although prolonged hypoxia transiently activates HIF-1α, persistent inflammation ultimately exacerbates vascular impairment (125). The interplay between macrophage-driven fibrosis and vascular damage constitutes the structural foundation of ovarian reserve depletion.

### 5.3 Primordial follicle pool dysregulation

The regulation of primordial follicle dormancy and activation is crucial for maintaining the ovarian reserve, with macrophages playing a pivotal role in this process.

#### 5.3.1 Induction of hyperactivation of primordial follicles

Pathologically activated M1 macrophages promote aberrant primordial follicle activation through the release of pro-inflammatory cytokines (126). Unlike growing follicles, primordial follicles lack a theca cell layer and consist solely of an oocyte surrounded by a single layer of flattened pregranulosa cells (127). Consequently, inflammatory mediators from M1 macrophages directly target pregranulosa cells or the quiescent oocyte itself, rather than acting via theca cell-mediated pathways (3, 36). Emerging evidence indicates that M1 macrophage-derived IL-6 and TNF-α activate the JAK/STAT3 signaling pathway (128), which in turn suppresses the expression of PTEN and relieves the inhibition of the PI3K/Akt axis (100, 129). This signaling cascade disrupts local quiescence cues, including the KIT ligand and Notch pathway inputs, thereby triggering premature recruitment from the dormant pool (130). However, without adequate growth factor support and a favorable microenvironment, the majority of these prematurely activated follicles fail to complete maturation and rapidly degenerate (131).

M1-driven inflammation produces two divergent fates for the primordial follicle pool. Excessive ROS/RNS inflicts irreversible oxidative damage, causing immediate atresia (36, 132). Conversely, sustained cytokine signaling via STAT3 and PI3K/Akt disrupts follicular quiescence, leading to accelerated recruitment and eventual burnout (66). The balance between these outcomes is dependent on two variables. First, spatial location: the collagen-dense, stiff, and hypoxic cortex renders follicles susceptible to HIF-1α-mediated inflammatory activation (63, 133), whereas the vascularized, oxygen-rich medulla predisposes them to ROS/RNS-induced oxidative atresia (134). Second, disease stage: early inflammaging, with modest ROS and dominant cytokine signaling, favors PI3K/Akt-dependent recruitment (131), whereas advanced aging, marked by accumulated oxidative damage and antioxidant depletion, shifts the balance toward direct oocyte injury and atresia (135). Currently, there is inadequate direct human clinical evidence to precisely define the threshold that regulates the transition from M1-induced oxidative atresia to hyperactivation-triggered follicular depletion. Urgent translational studies are required to confirm this immunometabolic hypothesis and to clarify the factors influencing follicular outcomes under conditions of ovarian inflammatory stress.

#### 5.3.2 Impaired efferocytic clearance

M2c macrophages maintain ovarian homeostasis through efferocytosis, a process mediated by a high MerTK expression that facilitates the clearance of apoptotic debris. Under pathological conditions, reduced MerTK levels or inadequate “eat me” signals (e.g., phosphatidylserine exposure) impair this clearance. Accumulated apoptotic fragments subsequently release DAMPs, including HMGB1 and mtDNA. These DAMPs activate pattern recognition receptors such as TLRs and the NLRP3 inflammasome, inducing the secretion of pro-inflammatory cytokines (TNF-α, IL-1β, and IL-6) (136). This establishes a self-amplifying cycle in which an impaired clearance permits DAMP accumulation, fueling persistent inflammation and accelerated tissue injury. Within this inflammatory milieu, macrophages undergo senescence and may transform into multinucleated giant cells or lipid-laden foam cells. These phenotypes are pro-inflammatory and pro-fibrotic, exacerbating the deterioration of the ovarian microenvironment.

Macrophage polarization imbalance contributes to ovarian reserve depletion through multiple interconnected mechanisms, including inflammation-induced oxidative stress, fibrosis, vascular dysfunction, and compromised efferocytosis (**Figure 2**). Although pharmacological targeting of NLRP3 has shown therapeutic potential in inflammatory and fibrotic disorders, its specific role in DOR requires further validation.

**Figure 2:**
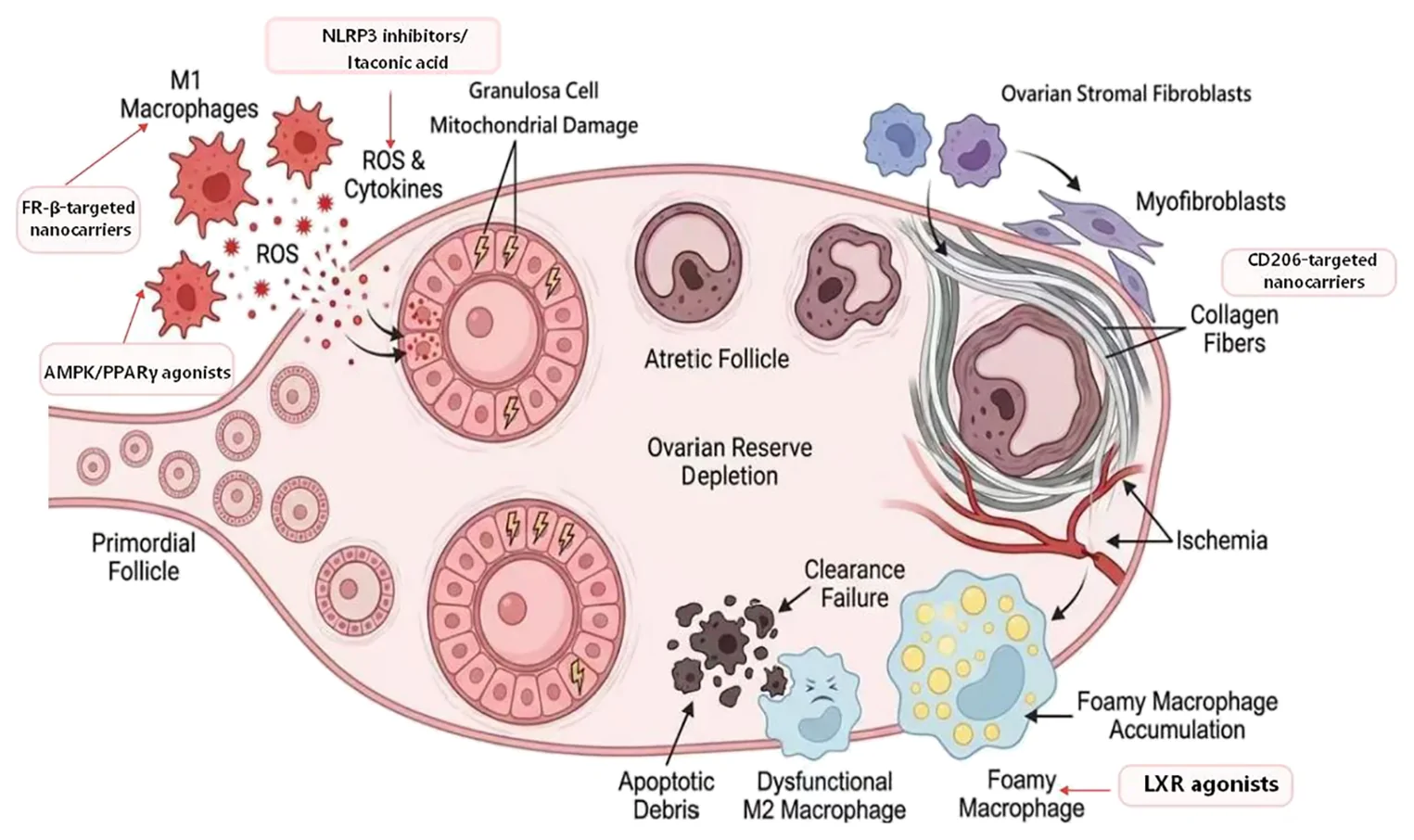
Schematic illustration of macrophage polarization imbalance driving ovarian reserve depletion. Pathologically activated M1 macrophages release reactive oxygen species (ROS) and pro-inflammatory cytokines, inducing mitochondrial damage and apoptosis in granulosa cells. Impaired clearance of apoptotic debris promotes foam cell accumulation. Concurrently, pro-fibrotic macrophages and the damaged microenvironment stimulate stromal fibroblast-to-myofibroblast differentiation, driving collagen deposition and ovarian stromal fibrosis. This fibrosis, together with vascular abnormalities, induces local ischemia, compromises the follicular microenvironment, and ultimately leads to follicular atresia. Corresponding therapeutic targets include AMP-activated protein kinase (AMPK)/peroxisome proliferator-activated receptor gamma (PPARγ) agonists to promote M1-to-M2 repolarization; folate receptor β-targeted nanocarriers for precise delivery to M1 macrophages and foam cells; NLRP3 inhibitors and anti-inflammatory agents to suppress ROS/cytokine axis-mediated pyroptosis; CD206-targeted nanocarriers against M2a pro-fibrotic macrophages; and liver X receptor (LXR) agonists to enhance the cholesterol efflux and prevent foam cell formation. This framework traces the pathological cascade from inflammatory initiation to functional decline, establishing a multi-target precision therapeutic strategy against the inflammation–fibrosis–reserve depletion axis in diminished ovarian reserve (DOR).

## 6 Therapeutic strategies targeting macrophage immune metabolism

Metabolic reprogramming of the macrophages in the DOR microenvironment drives pathological polarization and ovarian functional decline. Building on this insight, current therapeutic efforts target core metabolic regulators and signaling pathways that govern macrophage phenotype through two complementary strategies: 1) pharmacological intervention with small-molecule agents to correct metabolic dysregulation and 2) technological intervention using bioengineered platforms and targeted delivery systems to precisely reprogram ovarian macrophage function. The following sections discuss the underlying pathways and translational potential of these approaches.

### 6.1 Targeting macrophage metabolic dysregulation

Therapeutic strategies for macrophage metabolic dysregulation in DOR comprise two main approaches: restoring metabolic flexibility and correcting lipid metabolism disturbances.

#### 6.1.1 Activating the AMPK/PPARγ axis

Activation of AMP-activated protein kinase (AMPK) and PPARγ suppresses M1-associated glycolytic metabolism, restores OXPHOS, and promotes M2 polarization. AMPK facilitates FAO and mitochondrial biogenesis, whereas PPARγ upregulates M2 markers such as Arg-1 (137, 138). As a canonical AMPK activator, metformin shifts macrophage polarization toward M2, reduces M1 abundance, attenuates inflammation, and preserves the ovarian reserve in models of ovarian aging or chemotherapy, as reflected by increased follicle counts and decreased atresia (139). This macrophage-specific protection mirrors a broader metabolic deficit in DOR in which AMPK signaling is similarly suppressed in granulosa cells, leading to impaired glucose uptake, reduced TCA cycle activity, and diminished ATP production (140). Therefore, restoring the metabolic homeostasis among ovarian cell types represents a critical therapeutic objective.

Clinically, metformin is generally well tolerated. However, 20%–30% of patients experience dose-dependent gastrointestinal adverse effects, including nausea and diarrhea. These effects can be mitigated through gradual dose titration or the use of extended-release formulations. Although lactic acidosis is rare, its risk warrants caution in patients with renal or hepatic impairment. With long-term use, periodic monitoring of vitamin B12 levels is required.

#### 6.1.2 Clearing foamy macrophages

Restoring cholesterol efflux is essential for correcting lipid metabolic disturbances and foam cell accumulation in DOR. LXR agonists promote cholesterol efflux and attenuate foam cell formation by upregulating ABCA1 and ABCG1 (141, 142). However, their clinical utility is constrained by hepatic steatosis and hypertriglyceridemia, which stem from the LXR-mediated activation of hepatic SREBP-1c-dependent *de novo* lipogenesis (143). Because this pathway is central to macrophage lipid homeostasis (119), such systemic toxicities highlight the need for localized delivery approaches. Itaconic acid (IA), an endogenous immunometabolite generated via the IRG1 (Immunoresponsive Gene 1) pathway, represents an alternative strategy. IA exerts anti-inflammatory and antioxidant effects through the inhibition of succinate dehydrogenase (144). In LPS-induced DOR mouse models, IA protects ovarian function, increasing the follicle counts across developmental stages and improving the reproductive hormone levels. These benefits are mediated by the activation of the NRF2 pathway and the suppression of the NLRP3 inflammasome-driven pyroptosis (145). IA and its derivatives, including 4-octyl itaconate, show promise for modulating the ovarian immunometabolic microenvironment in DOR. Nevertheless, clinical evidence in humans remains limited and requires further investigation (146).

### 6.2 Engineered delivery systems

While small-molecule drugs show therapeutic potential, their systemic administration is limited by poor tissue targeting, off-target effects, and inadequate local accumulation in the ovaries. Engineered delivery platforms overcome these challenges and enable precise local intervention.

#### 6.2.1 Metabolic-targeting engineered exosomes

Disruption of ovarian immunometabolic homeostasis is closely linked to DOR. Single-cell sequencing analyses have identified significant cellular alterations in aging ovaries (147). A 2024 human ovarian single-cell atlas revealed a pro-inflammatory macrophage subset, termed apoptotic macrophages, within the inflammatory microenvironment: this subset drives stromal aging and reproductive decline (148). Murine studies further confirm a distinct macrophage subpopulation (CX3CR1^low^CD81^high^) in aged ovaries, accompanied by altered annexin and TGF-β signaling (104). Collectively, these findings suggest that macrophage metabolic dysregulation may represent a nexus linking intercellular communication defects to ovarian dysfunction.

Mesenchymal stem cells (MSCs) (95) and their secreted exosomes (149) show translational promise as metabolic regulatory messengers. Exosomes can reprogram macrophage metabolism by transferring metabolism-related miRNAs, functional mitochondria, or signaling proteins. Recent studies demonstrate that small extracellular vesicles derived from avian MSCs—characterized by uniform size, high stability, and enriched miR-21-5p—enhance granulosa cell function, reduce apoptosis, and promote steroidogenesis in premature ovarian insufficiency (POI) models (150). These specialized vesicles outperform endogenous exosomes.

Nevertheless, the safety profile of exosome-based therapies remains incompletely characterized. Key concerns include immunogenicity, batch-to-batch variability, off-target cell activation, and potential oncogenicity. The prolonged retention of exosomes in the ovarian microenvironment and their unintended systemic distribution also remain unresolved. Furthermore, scalability, manufacturing consistency, and cost-efficiency pose practical barriers to their clinical translation, necessitating stringent quality control and comprehensive toxicological evaluation.

#### 6.2.2 Intelligent nanosystems for targeted delivery

Nanotechnology can substantially improve the pharmacokinetic profiles of metabolism-modulating therapies. Surface engineering with macrophage-specific ligands, such as those targeting the mannose receptor (CD206) (151) or folate receptor beta (152), enables precise macrophage-directed delivery in diverse disease models (153). CD206 is predominantly expressed on M2 macrophages, which mediate anti-inflammatory and reparative responses. This receptor therefore represents an ideal target for the delivery of therapeutics to the pro-fibrotic M2a subset implicated in ovarian fibrosis (154). In contrast, folate receptor beta is selectively upregulated on activated M1 macrophages and foam cells, providing a distinct target for the modulation of the pro-inflammatory and oxidative pathways (155, 156). Targeted nanocarriers directed against these receptors could thus transport small molecules (e.g., LXR agonists or itaconate derivatives) or nucleic acid therapeutics (e.g., siRNA) to disease-associated macrophages within the ovarian microenvironment. Responsive nanocarriers engineered to react to local oxidative stress or acidic pH have demonstrated on-demand drug release in preclinical models (157, 158). A recent study showed that tuning the shape, size, and surface chemistry of vasoactive intestinal peptide (VIP)-based amphiphilic micelles enhances macrophage binding and uptake (159), providing a rationale for VIP-based nanotherapeutics targeting ovarian macrophages.

Nevertheless, safety and manufacturing barriers hinder the clinical translation of nanocarriers. Safety concerns include off-target accumulation, immunogenicity, and poorly understood ovarian clearance kinetics (160). Practical challenges include batch-to-batch reproducibility, formulation stability, and cost-effectiveness. Premature drug release and degradation product formation can produce off-target toxicity, highlighting the need for comprehensive pharmacokinetic and toxicokinetic evaluation in preclinical models prior to clinical translation (161).

## 7 Discussions and perspectives

This review highlights the central role of macrophage polarization imbalance driven by metabolic reprogramming in the pathogenesis of DOR. This perspective marks a shift from the conventional oocyte-centric (“seed”) view to recognition of microenvironmental (“soil”) dysregulation within the ovarian immune microenvironment. Metabolic reprogramming drives pathological macrophage polarization and functionally links the inflammatory microenvironment to declining ovarian function. Consequently, therapeutic strategies targeting macrophage metabolism and polarization may shift the management of DOR from symptomatic relief to etiological intervention and from systemic immunomodulation to precision immunometabolic reprogramming.

Pharmacological modulators—including AMPK/PPARγ activators and LXR agonists—and bioengineered delivery platforms such as exosomes and targeted nanocarriers show preclinical promise for DOR. To accelerate clinical translation, future research should address four interconnected priorities. Firstly, studies must move beyond phenomenological descriptions to establish causal mechanisms. Macrophage-specific gene-edited models, combined with spatial multi-omics and metabolic flux analysis, are needed to determine whether specific metabolic shifts (e.g., enhanced glycolysis or reduced FAO) are necessary and sufficient for DOR pathogenesis. Secondly, metabolic crosstalk within the ovarian microenvironment requires systematic mapping. Co-culture systems, organoid models, and extracellular vesicle profiling can define how macrophages bidirectionally communicate with granulosa cells, oocytes, and stromal cells through metabolic intermediates, cytokines, and non-coding RNAs to maintain homeostasis or drive pathology. Thirdly, precision therapeutics warrant continued refinement. Novel small molecules that selectively reprogram macrophage metabolism and dual-targeting delivery systems directed at both ovarian tissue and macrophage subsets should be developed to maximize efficacy while minimizing systemic exposure. Finally, translational tools require optimization. Animal models that faithfully recapitulate human DOR progression and high-dimensional omics platforms for the identification of dynamic, noninvasive serum biomarkers of ovarian macrophage function are essential for early diagnosis, patient stratification, and treatment monitoring.

In conclusion, macrophages serve as central integrators of ovarian immunity and metabolism. Their functional reprogramming yields mechanistic insights and therapeutic targets for DOR. Synthesizing advances in immunology, metabolomics, bioengineering, and reproductive medicine—and systematically advancing the research priorities outlined above—will translate the macrophage-centered immunometabolic axis into clinical strategies that delay ovarian functional decline and improve reproductive outcomes.
